# A Combination of Cytokines Rescues Highly Purified Leukemic CLL B-Cells from Spontaneous Apoptosis *In Vitro*

**DOI:** 10.1371/journal.pone.0060370

**Published:** 2013-03-26

**Authors:** Hussein Ghamlouch, Hakim Ouled-Haddou, Gandhi Damaj, Bruno Royer, Brigitte Gubler, Jean-Pierre Marolleau

**Affiliations:** 1 EA4666, Laboratoire d′Immunologie, Université de Picardie Jules Verne, UFR de Médecine, Amiens, France; 2 Laboratoire d′Oncobiologie Moléculaire, CHU d′Amiens, Amiens, France; 3 Service d′Hématologie Clinique et Thérapie Cellulaire, CHU d′Amiens, Amiens, France; Westmead Millennium Institute, University of Sydney, Australia

## Abstract

B-chronic lymphocytic leukemia (B-CLL), the most common human leukemia, is characterized by predominantly non-dividing malignant mature CD5+ B lymphocytes with an apoptosis defect. Various microenvironmental stimuli confer a growth advantage on these leukemic cells and extend their survival *in vivo*. Nevertheless, when cultured *in vitro*, CLL B-cells rapidly die from apoptosis. Certain cytokines may extend the survival capacity of CLL B-cells *in vitro* and individual anti-apoptotic effects of several cytokines have been reported. The potential cumulative effect of such cytokines has not been studied. We therefore investigated the effects on CLL B-cells survival *in vitro* of humoral factors, polyclonal lymphocyte activators and a combination of cytokines known for their anti-apoptotic effects. Purified CLL B-cells were cultured in the presence or absence of various soluble molecules and the leukemic cell response was assessed in terms of viability. Apoptotic cell death was detected by flow cytometry using annexinV and 7-amino-actinomycin. The survival of CLL B-cells *in vitro* was highly variable. When tested separately, cytokines (IL-2, -6, -10, -12, -15, -21, BAFF and APRIL) improved CLL B cell survival moderately; in combination, they significantly enhanced survival of these cells, even up to 7 days of culture. We also report that humoral factors from autologous serum are important for survival of these malignant cells. Our findings support the concept that the CLL microenvironment is critical and suggest that soluble factors may contribute directly to the prolonged survival of CLL B-cells. Therefore, the combination of cytokines we describe as providing strong resistance to apoptosis *in vitro* might be used to improve the treatment of CLL.

## Introduction

B-chronic lymphocytic leukemia (B-CLL), the most common human leukemia, involves the accumulation of predominantly quiescent CD5+ B cells driven by the proliferation of a subpopulation [1]. In addition to this atypical proliferative profile, the B-CLL cells exhibit a strong dependence on cellular and cytokine components of their microenvironment, making their manipulation *ex vivo* complex and resulting in biased findings [2]. Indeed, B-CLL can affect diverse tissues by the infiltration of malignant cells into blood compartments, bone marrow and lymph nodes. The B-CLL cells isolated from these tissues exhibit heterogeneity in their transcriptional, phenotypic, proliferative and apoptotic profiles dictated by the local microenvironments [3], [4], [5], [6]. Studies of these tissues and the extrapolation from our knowledge of normal B cell biology [7] suggest that the lymph node is the seat of B-CLL pathogenesis. However, for reasons of inaccessibility and ethics, almost all relevant scientific studies have addressed peripheral blood leukemic cells, only partially reflecting B-CLL physiopathology. Research is further impeded by the absence of a cellular model for B-CLL.

The most explicit demonstration of the influence of the microenvironment on B-CLL is the spontaneous apoptosis of B-CLL cells cultured *ex vivo*. This spontaneous apoptosis can be prevented by the presence of accessory cells and cytokines [2], [8]. Several modulators of the survival of CLL cells have been identified and include soluble factors and cell-cell interactions [9], [10]. Nurse-like cells [11], bone marrow stromal cells [12], [13], T cells [14], [15], dendritic cells [16], accessory leukocytes [17], the leukemic cells themselves [18] and the cytokines derived from them: BAFF and APRIL [19], IL-6 [20], IL-10 [21], IL-2 [22], IL-4 [23], IL-21 [24], IL-12 [25] and IL-15 [26], [27], have all been suggested to act as pro-survival factors or growth factors in CLL. Nevertheless, it is unclear whether all the cytokines that promote viability *in vitro* also play a significant role *in vivo*; it is consequently relevant to study the effect of humoral components on CLL B-cell survival [28], [29]. Antigenic stimulation is the initial molecular event triggering the expansion of B lymphocytes. Therefore, in view of the role of the B-cell receptor in normal B cell physiology, it is possible that the antigenic stimulation of B-CLL cells is an oncogenic event leading to the proliferation and survival of a leukemic clone [30]. Indeed, signaling via the B-cell receptor may activate proliferation and survival pathways in CLL cells [31].

These various stimuli are provided by diverse components of the microenvironment where CLL cells accumulate, and are believed to extend the survival capacity of B-CLL cells *in vitro*. A major goal in CLL therapy is both to induce apoptosis in leukemic cells and to overcome their resistance to apoptosis. The CLL microenvironment makes an important contribution to increasing resistance to apoptosis. *In vitro* studies evaluating the efficacy of treatment for CLL can help identify possible therapeutic strategies [32]. We therefore investigated the effects of a combination of cytokines and the effects of humoral factors and antigenic stimulation on apoptosis of B-CLL cells *in vitro*. Partial re-creation *in vitro* of the B-CLL microenvironment, including in particular leukemic cell survival signals, is a potentially promising approach for testing existing and new treatments to overcome resistance to apoptosis.

## Materials and Methods

### Patients

Leukemic lymphocytes were obtained from the peripheral blood of 15 B-CLL patients (9 women and 6 men, with median age 74 (54–80) years), diagnosed according to the international guidelines (Table S1). All patients provided written informed consent. All studies involving patient samples were approved by the local ethics committee (Comité de Protection des Personnes (CPP) Nord-Ouest, France). All cases involved clonal expansion of small lymphocytes with high nucleus/cytoplasm ratios that co-expressed CD19, CD5, and CD23. None of the patients were being treated at the time of the analysis.

### Cell isolation and culture

Peripheral blood mononuclear cells (PBMC) were isolated from heparinized venous blood from CLL patients by Ficoll density gradient centrifugation. CD19+ CD5+ CLL B-cells were purified by negative selection using Magnetic Bead-Activated Cell Sorting (MACS), with a B cell (B-CLL) isolation kit (MiltenyiBiotec). The purity of all preparations was always about 98% and the cells co-expressed CD19, CD20 and CD5 on their cell surfaces, as assessed by flow cytometry. For cell culture, 10^6^ purified B-CLL cells/well were cultured in 24-well plates in 1 ml of RPMI 1640 medium (Gibco-Invitrogen) supplemented with fetal calf serum (FCS, PAA) 100 IU/mL penicillin, 100 μg/mL streptomycin and 2 mM L-glutamine at 37°C in a 5% humidified incubator. The cells were incubated, from the initiation of cultures, with various molecules: IL-2 (50 ng/ml), IL-4 (50 ng/ml), IL-6 (50 ng/ml), IL-10 (10 ng/ml), IL-12 (10 ng/ml), IL-15 (10 ng/ml), IL-21(10 ng/ml), BAFF (10 ng/ml), APRIL (10 ng/ml), phorbol myristate acetate (PMA, 1 µg/ml) (Santa Cruz Biotechnology), phytohemagglutinin (PHA, 5 µg/ml) (Sigma-Aldrich), ionomycin (0.1 µg/ml) (Santa Cruz Biotechnology), lipopolysaccharide (LPS, 1 µg/ml), autologous patient serum (AS), heterologous serum (HS) from healthy donors, and a cocktail of cytokines (Cc) which included IL-2, IL-6, IL-10, IL-12, IL-15, IL-21, BAFF and APRIL. Cells in complete medium were used as controls. All cytokines were purchased from PeproTech EC.

### Cell viability

Cell viability was measured by flow cytometry using annexin V–phycoerythrin (PE) and 7-amino-actinomycin (7-AAD) staining. Cells were harvested and resuspended in 100 µl annexin V–binding buffer containing 5 µl Annexin V–PE and 5 µl 7-AAD staining solutions (BD Biosciences), incubated for 15 minutes at RT, and analyzed in a FACSCantoII or FACSAriaII flow cytometer (BD Biosciences). FlowJo software (Tree Star) was used for data analysis. AnnexinV- 7AAD- double-negative cells were counted as viable. The results were confirmed by determining changes in forward light scattering properties associated with dead cells, which were smaller than viable cells.

### Statistical analysis

Prism 5 software (GraphPad Software) was used for statistical analyses. The statistical significance of differences between groups was determined using Student's t test or the Wilcoxon test, as appropriate; P values less than 0.05 (0.01) were considered (highly) statistically significant, ^*^ P<0.05, ^**^P<0.01.

## Results

### Autologous serum (AS) protects CLL B-cells from apoptosis *in vitro*

**Figure 1:**
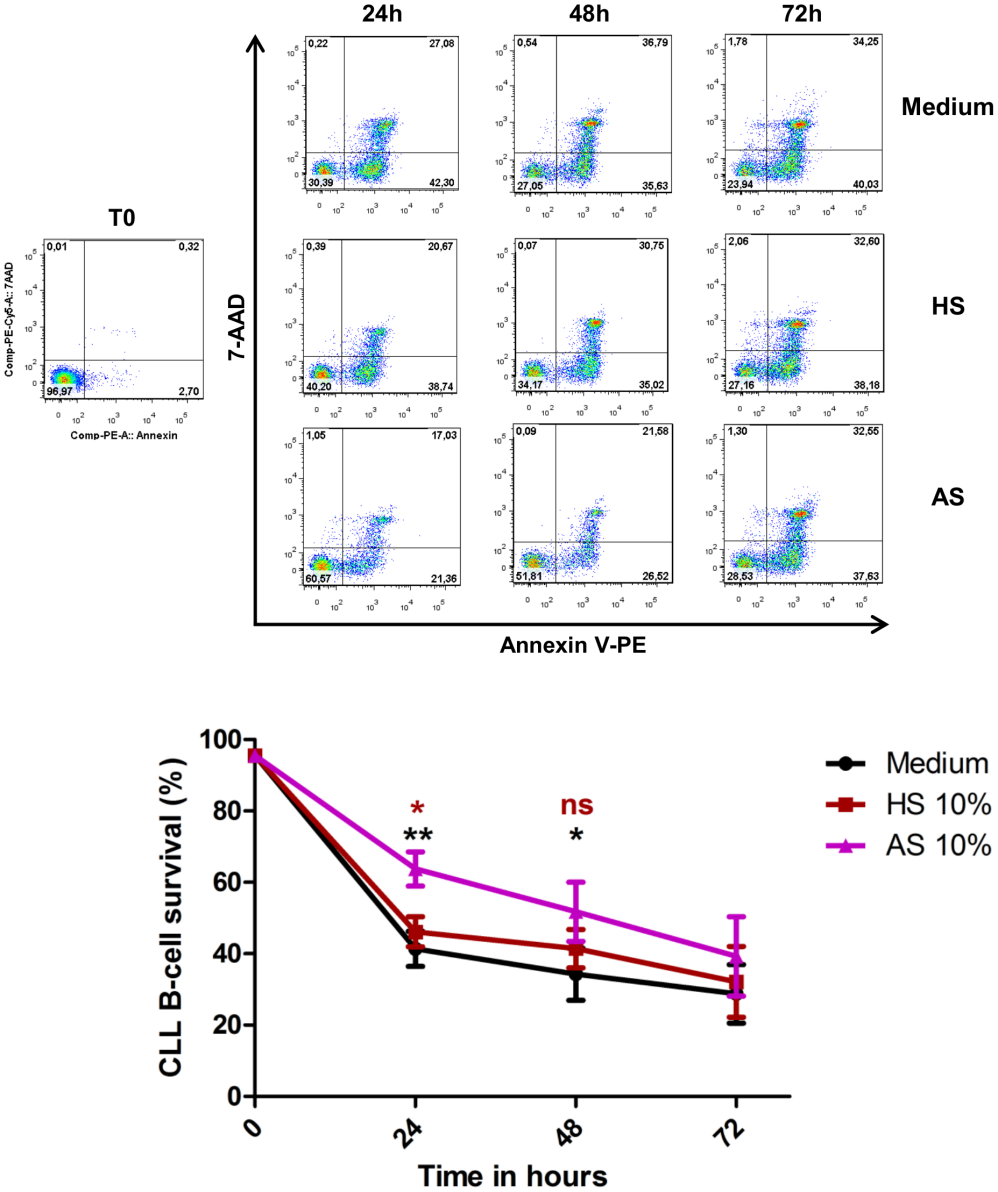
Autologous serum reduced spontaneous apoptosis of CLL B-cells *in vitro*. Cells were incubated in the presence of 10% AS, 10% HS or complete medium and cell viability was determined after 24, 48 and 72 hours of culture by annexin V–PE/7-AAD staining and flow cytometry. Upper panel: Cytometry plots from a representative patient. Lower panel: The values reported are means ± SEM for 8 independent experiments, each performed in duplicate. Significance was calculated with the Wilcoxon test: ^*^p<0.05 ^**^p<0.01. Black asterisks are for AS versus Medium, and brown asterisks for AS versus HS.

To determine the effects of AS on CLL B-cells survival we incubated cells in the presence of 10% of AS. Complete medium and medium supplemented with 10% heterologous serum (HS) were used as controls (Figure 1). The percentages of viable CLL B-cells after 24 h were highly significantly greater in the presence of AS (63.7%±13.4%) than HS (46.1%±11.1%) or in complete medium (41.3%±13.8%) After 48 h of incubation, the difference between AS (51.7%±23.3%) and complete medium (34.2%±20.7%) remained significant, and the viability in HS conditions was intermediate (41.4%±14.2%; not significant) (Figure 1). After 72 h of culture, viability in the presence of AS was not significantly higher than in the other two conditions (Figure 1).

### Effect of cytokines on survival of CLL B-cells *in vitro*

**Figure 2:**
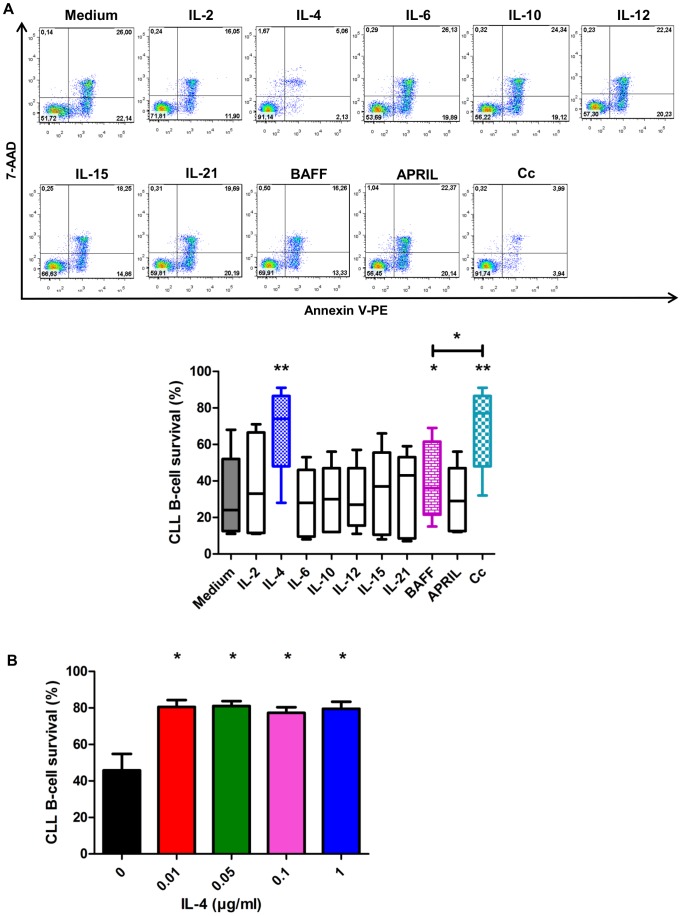
IL-4 and Cytokine cocktail increases survival of B-CLL cells *in vitro*. **A.** Cytokines were added to the cultures and the survival of B-CLL cells was analyzed after 72 hours of culture by annexin V–PE/7-AAD staining and flow cytometry. Upper panel: Cytometry plots from a representative patient. Lower panel: The percentages of CLL B-cells surviving in 5 independent experiments (for IL-2, IL-6, IL-10, IL-12, IL-15, IL-21, BAFF and APRIL) and 9 independent experiments for IL-4 and Cc are represented as box and whisker (Min to Max) plots. The significance of differences was calculated with the Wilcoxon test: ^*^p<0.05 ^**^p<0.01. **B.** Cells were incubated for 48 h with IL-4 concentrations of 0.01 to 1 µg/ml. The values reported are means ± SEM for 4 independent experiments, each performed in duplicate. The significance of differences was calculated with the paired t-test: ^*^p<0.05.

The effects of nine cytokines (IL-2,-4,-6,-10,-12,-15,-21, BAFF and APRIL) on CLL B-cells survival was determined (Figure 2A, Table S2). Only IL-4 (67.1%±21.7%) and BAFF (40.4%±21.3%) had significant effects on survival. All other cytokines caused non-significant increases of CLL B-cell survival: the percentages of viable cells were 32.2%±21% in complete medium (control), and 37.8%±27.8%, 29.6%±18.6%, 29.4%±18.2%, 30.2%±17.2%, 33.8%±23.8%, 33.2%±23.3%, and 29.4%±18% in the presence of IL-2, IL-6, IL-10, IL-12, IL-15, IL-21 and APRIL, respectively. However, the presence of a combination of all these cytokines, except IL-4, resulted in a highly significant survival effect: viability was 67.3%±21% (Figure 2A). Samples were also incubated in the presence of each of a series of concentrations of IL-4 (Figure 2B), and the survival effect of IL-4 did not differ significantly between the concentrations used.

### PMA increases survival of CLL B-cells *in vitro*

**Figure 3:**
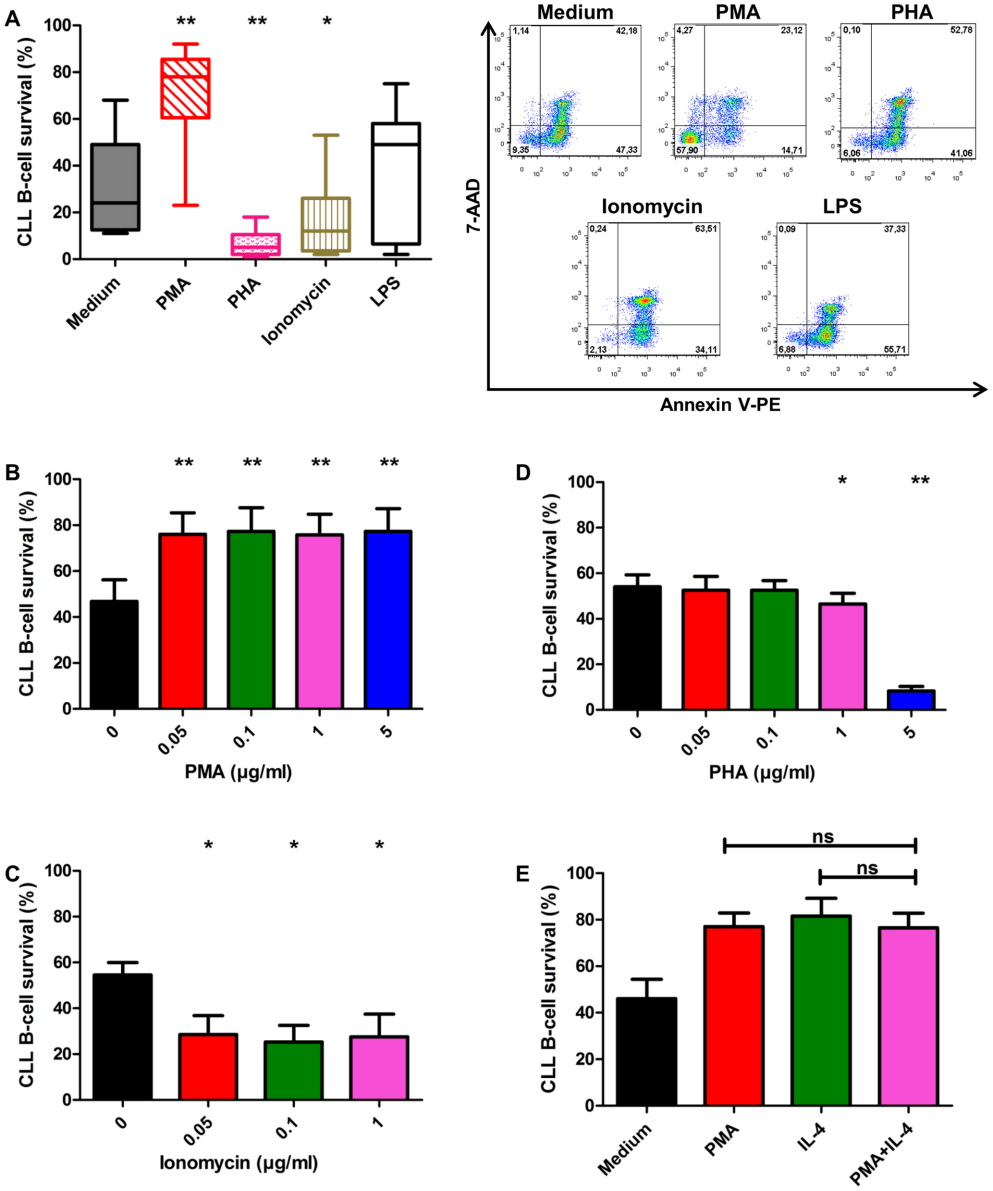
PMA increases survival of CLL B-cells *in vitro* at all doses tested. **A.** After 72 h, the survival of B-CLL cells in the presence of PMA (1 µg/ml) was significantly greater than that of control cells in complete medium as evaluated by annexin V–PE/7-AAD staining. Exposure to LPS (1 µg/ml) had no effect on cell survival. Right panel: Cytometry plots from a representative patient. Left panel: The presence of PHA (5 µg/ml) or ionomycin (0.1 µg/ml) significantly decreased cell survival. The percentages of CLL B-cells surviving after 72 hrs in 9 independent experiments are presented as box and whisker (minimum to maximum) plots. The significance of differences was calculated with the Wilcoxon test: ^*^p<0.05 ^**^p<0.01. **B.** Cell survival after incubation for 48 h with each of a series of concentrations of PMA (0.05 to 5 µg/ml). **C.** Cell survival after 48 h of incubation in the presence of 0.05, 0.1 and 1 µg/ml ionomycin. **D.** Cell survival after incubation for 48 h with 0.05 to 5 µg/ml PHA. **E.** Survival of CLL B-cells after 48 h of exposure to a combination of 1 µg/ml PMA and 0.01 µg/ml IL-4. In B, C, D, E, results are represented as means ± SEM for 4 independent experiments, each performed in duplicate. The significance of differences was calculated with the paired t-test: ^*^p<0.05 ^**^p<0.01.

We tested for the effects of antigenic stimulation on CLL B-cell survival. Cells were cultured with the following polyclonal stimulators: PMA, PHA, ionomycin and LPS (Figure 3A). In the presence of 1 µg/ml of PMA, the number of viable CLL B-cells (70.7%±20.9%) was significantly higher than that in complete medium (32.2%±21%). LPS had no major effect on CLL B-cell survival (35.1%±28.4%). Surprisingly, each ionomycin and PHA significantly induced apoptosis of CLL B-cells: the percentages of viable cells were 17.6%±16.6% and 6.6%±5.9%, respectively. We analyzed the dose effect relationships for PMA, PHA, and ionomycin. In our experiment, the pro-survival effect of PMA and the pro-apoptotic effect of ionomycin appeared to be independent of dose used (Figure 3B, 3C). By contrast, PHA significantly increased apoptosis from a dose of 1 µg/ml (Figure 3D). CLL B-cells were treated with a combination of PMA and IL-4 to test for any cumulative effect on survival (Figure 3F): the effect of this combination on CLL B-cells survival was no greater than either of the individual effects.

### PMA, IL-4 and Cc have lasting pro-survival effects

**Figure 4:**
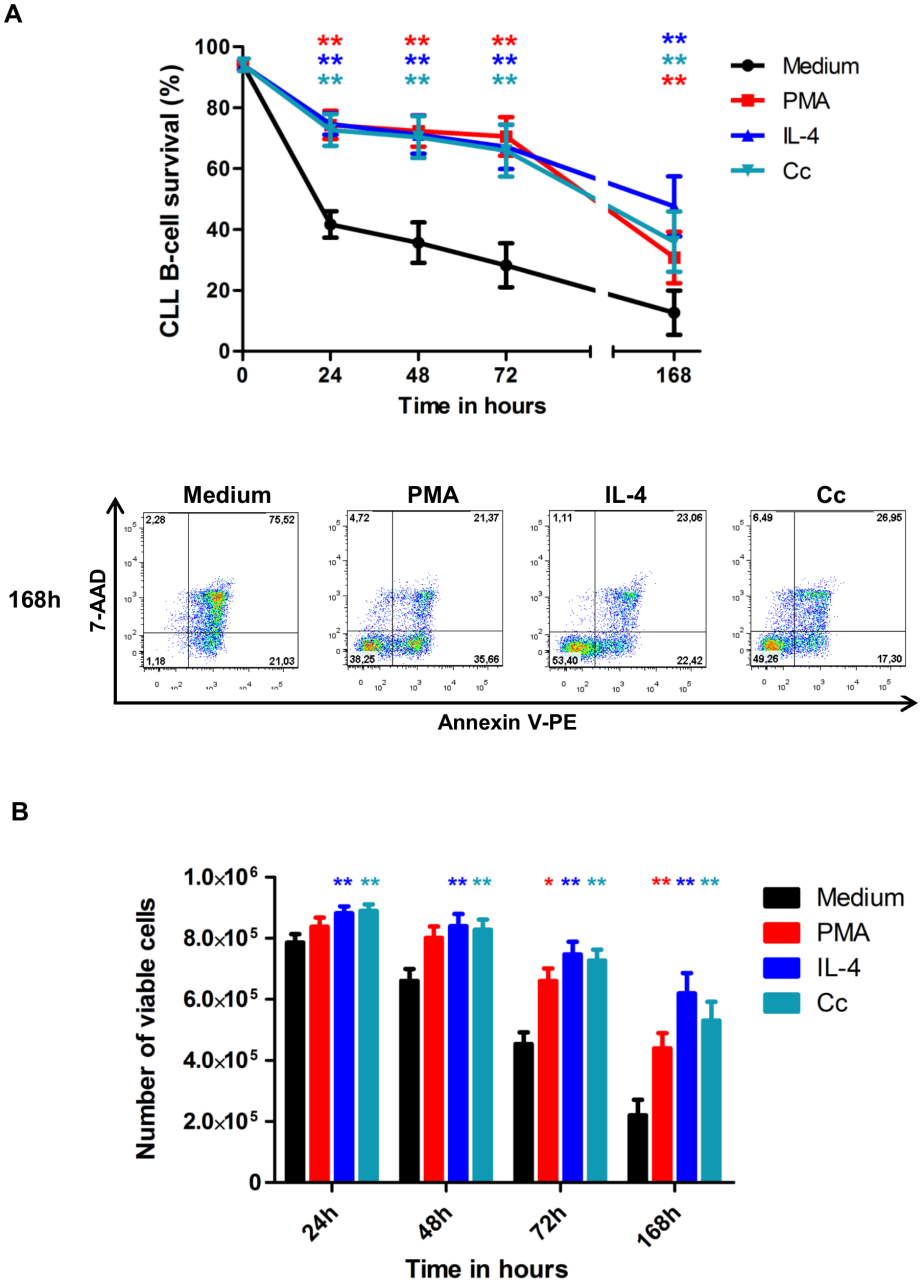
The pro-survival effect of PMA, IL-4 and a cytokine cocktail is sustained for 7 days of culture. **A.** Apoptosis was evaluated after 24, 48, 72 and 168 hours of culture by annexin V–PE/7-AAD staining and flow cytometry. The survival of B-CLL cells in the presence of PMA or IL-4 or cytokine cocktail was greater than that of controls for up to 168 h. Lower panel: Cytometry plots from a representative patient at 168 h. Upper panel: The values reported are means ± SEM for 9 independent experiments, each performed in duplicate. **B.** 10^6^ purified B-CLL cells/well were cultured in 24-well plates in 1 ml of RPMI 1640 complete medium in the presence of PMA or IL-4 or the cytokine cocktail. Changes in viable cell number were assessed (counted in duplicate) by a trypan blue exclusion method after 24, 48, 72 and 168 hours of culture. The values reported are means ± SEM for 9 independent experiments. The significance of differences was calculated with the Wilcoxon test: ^*^p<0.05 ^**^p<0.01. Red asterisks for PMA versus Medium, clear blue asterisks for Cc versus Medium and indigo asterisks for IL-4 versus Medium.

To assess whether the pro-survival effects of PMA, IL-4 and Cc were sustained, apoptosis was checked after 24 h, 48 h, 72 h and 168 h (Figure 4). After 168 h, the percentages of viable cells were 30.7%±25.3% in the presence of PMA, 47.5%±29.5% in that of IL-4 and 36%±29.7% in that of Cc, all significantly higher than the control value of 12.6%±21.8% in complete medium. Changes in viable cell number are represented in Figure 4B. In the complete medium controls, the increases of apoptosis over time caused a substantial reduction in cell number. By contrast, the diminished apoptosis of PMA-, IL-4- and cytokine combination-treated cells resulted in a significant preservation of the numbers of viable cells (Figure 4B).

## Discussion

**Figure 5:**
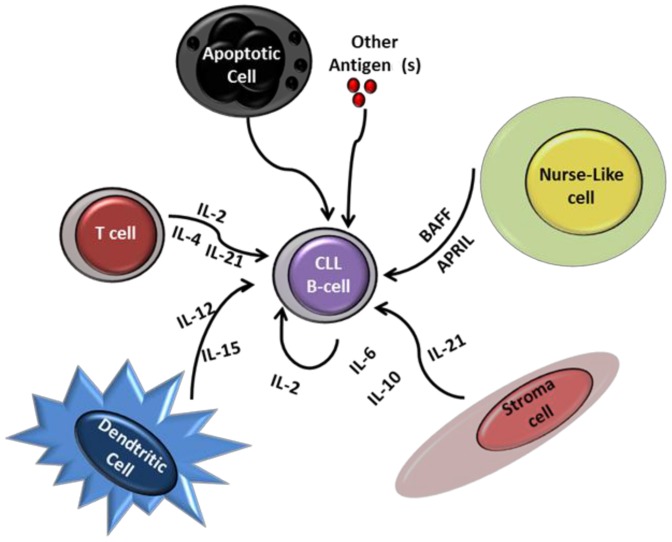
CLL B-cells migrate from the peripheral blood to lymph nodes or bone marrow to receive the appropriate signals for their growth and survival. The peripheral blood of CLL patients contains various cytokines that can protect CLL B-cells from apoptosis. When CLL B-cells travel into lymph nodes or bone marrow, they make contact with various cells in the microenvironment (dendritic cells, stromal cells, T cells and Nurse-like cells), the cytokines produced by them, and various antigens that are able to promote CLL B-cell survival.

Programmed cell death or apoptosis is a common form of cell elimination that can be activated in different cell types in response to a number of physiologically relevant stimuli. *In vivo*, CLL CD5+ B-cells are arrested in G0 and display enhanced survival, whereas they undergo spontaneous apoptosis *in vitro*. This suggests the existence of apoptosis-inhibitory factors or survival-support factors *in vivo* that may be involved in the accumulation of these cells. Such factors may be provided by cells in the CLL microenvironment and by B-CLL cells themselves. It is currently believed that CLL B-cells migrate several times from the peripheral blood to lymph nodes or to bone marrow where they receive signals for their growth and survival. Although the mechanisms are not known, CLL B-cells express a set of chemokine receptors regulating their trafficking between different tissues [33]. Numerous cytokines, including IL-2, IL-4, IL-6, IL-10, IL-12, IL-15, IL-21, BAFF and APRIL individually or in some cases in combination, have been implicated in the regulation of B-CLL cell apoptosis *in vitro* [8], [9], [19], [20], [21], [22], [24], [25], [27]. The cellular B-CLL microenvironment is constituted of various types of cells able to produce and secrete all of these cytokines (Figure 5). However, the effect of the combination of all these cytokines on CLL B-cell apoptosis *in vitro* has not been studied. Consistent with earlier studies, we observed that IL-4 and BAFF each had significant pro-survival effects. In our experimental conditions, IL-2, IL-6, IL-10, IL-12, IL-15, IL-21 and APRIL failed to enhance cell viability significantly. Nevertheless, we show that a combination of these cytokines significantly increased CLL B-cell survival (Figure 2). The effect of Cc on CLL B-cells was comparable to that of IL-4. This agrees with the findings that soluble factors provided by the microenvironment increase the growth and survival capacity of leukemic cells. Our study clearly indicates that these factors might act in synergy to protect CLL cells from apoptosis. Differential expression of cytokine receptors by CLL B-cells of the same clone may explain the lesser effect on survival of individual cytokines, and, at least, the pro-survival effect of Cc.

By contrast to the well-documented pro-survival effect of phorbol ester on B-CLL cells [34], the effects of other polyclonal activation molecules, and in particular ionomycin, PHA and LPS, are not well documented. Consistent with earlier studies [35], we showed that PMA has a significant pro-survival effect on CLL B-cells, and that this effect did not differ between the doses we tested (Figure 3). This pro-survival effect of PMA is mediated by the activation of the PKC but not MAPK pathway [34]. Unexpectedly, PHA and ionomycin induced apoptosis in CLL B-cells (Figure 3). No such pro-apoptotic effect of these molecules has been reported before, possibly because these molecules are often used in combination with phorbol ester which may mask this effect (data not shown). However, dose-response experiments showed that ionomycin is pro-apoptotic at all doses tested, and that PHA has no effect on cell survival at concentrations of 0.05 and 0.1 µg/ml but had a significant pro-apoptotic effect at 1 and 5 µg/ml (Figure 3). The concentrations of several cytokines including IL-2, IL-4, IL-6, IL-10, IL-12, BAFF and APRIL, is abnormally high in the serum of CLL patients [10], [36], [37], [38]. Coherent with this observation, we show, for the first time, that autologous serum reduces apoptosis of highly purified CLL B-cells (Figure 1). The pro-survival effects of PMA, IL-4 and the cytokine cocktail on CLL B-cells continued for at least 7 days of culture (Figure 4). These various findings contribute to our understanding of how the B-CLL microenvironment supports the survival of B-CLL cells; in particular, CLL B-cells appear to migrate several times between peripheral blood and lymph nodes/bone marrow where they receive growth and survival signals. It is also now clear that the CLL microenvironment plays a crucial role in resistance of CLL cells to therapy [32], [39], [40].

In conclusion, our data show that spontaneous apoptosis of B-CLL cells incubated with autologous serum was significantly lower than that of control CLL B-cells (incubated with heterologous serum or complete medium). Moreover, we report that a combination of several cytokines significantly enhances and prolongs the survival of these cells *in vitro*. Therefore, our findings support the notion that autocrine (B-CLL cell-derived cytokines) and paracrine (IL-2, IL-4, IL-6, IL-10, IL-12, IL-15, IL-21, BAFF and APRIL from T cells, stromal cells, dendritic cells and Nurse-like cells) factors (Figure 5), and subsequent growth loops established by interactions of such cytokines may make large contributions to the progressive accumulation of B-CLL cells. The combination of cytokines we describe, and that provides substantial resistance to apoptosis *in vitro*, may be used to improve CLL therapy. Also, the ability to interrupt such cytokine networks *in vivo* may be exploited to amend existing therapies for B-CLL.

## Supporting Information

Figure S1 **Flow cytometry analysis of CLL B-cell purity.** CLL B-cells were obtained from peripheral blood of CLL patients after negative selection with the B-Cell (B-CLL) isolation kit (Miltenyi). Upper plot: Left dot plot shows forward scatter (FSC) and side scatter (SSC) parameters. Middle plot shows IgG1-FITC and IgG1-Percp-Cy5.5 isotype controls. Right plot shows purity percentage as evaluated by double staining the cells with anti-CD19-FITC and anti-CD5-Percp-Cy5.5 antibodies (BD Biosciences). Lower histograms: cells were systematically checked for contamination by labeling with anti-CD2-FITC, anti-CD14-PE and anti-CD56-APC antibodies. Black line: Isotype control, Blue line: CD2, CD14 or CD56 expression.
(TIF)

Table S1 **Patient characteristics.**
(TIF)

Table S2 **Survival levels of CLL B-cells in response to cytokines at 72 h.** The percentages of survival in vitro of CLL B-cells from 5 patients in the presence of IL-2, IL-6, IL-10, IL-12, IL-15, IL-21, BAFF and APRIL and from 9 patients in the presence of IL-4 and Cc were determined by annexin V and 7-AAD staining. The values reported are the annexin V/7-AAD double negative population as a percentage of the total population.
(TIF)
