# Supplementary figures and images for: A Combination of Cytokines Rescues Highly Purified Leukemic CLL B-Cells from Spontaneous Apoptosis In Vitro
Source: PLoS One. 2013 Mar 26;8(3):e60370. doi: 10.1371/journal.pone.0060370 (PMC3608602; doi:10.1371/journal.pone.0060370)

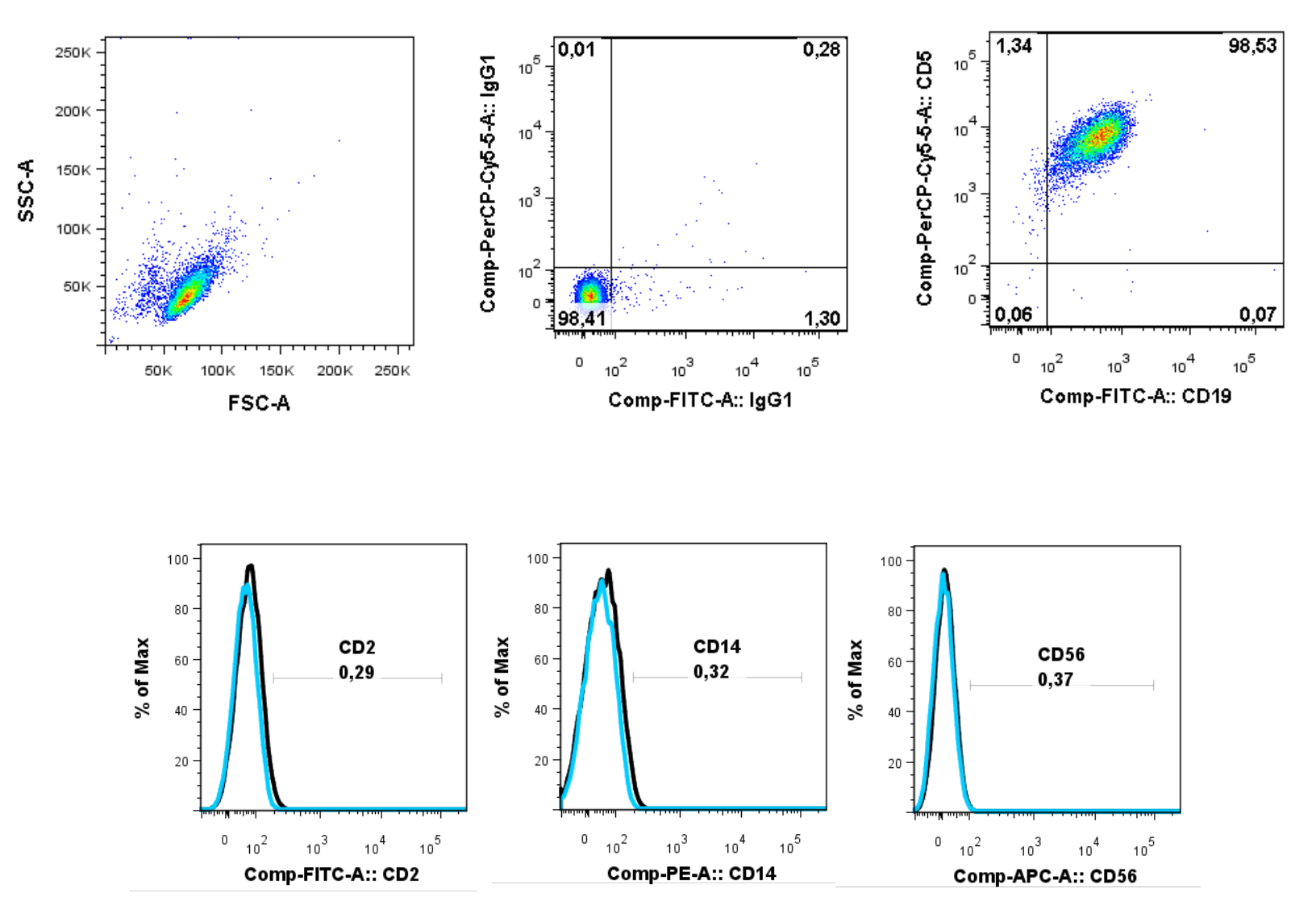

Supplement: Figure S1 — Flow cytometry analysis of CLL B-cell purity. CLL B-cells were obtained from peripheral blood of CLL patients after negative selection with the B-Cell (B-CLL) isolation kit (Miltenyi). Upper plot: Left dot plot shows forward scatter (FSC) and side scatter (SSC) parameters. Middle plot shows IgG1-FITC and IgG1-Percp-Cy5.5 isotype controls. Right plot shows purity percentage as evaluated by double staining the cells with anti-CD19-FITC and anti-CD5-Percp-Cy5.5 antibodies (BD Biosciences). Lower histograms: cells were systematically checked for contamination by labeling with anti-CD2-FITC, anti-CD14-PE and anti-CD56-APC antibodies. Black line: Isotype control, Blue line: CD2, CD14 or CD56 expression. (TIF) [file pone.0060370.s001.tif]

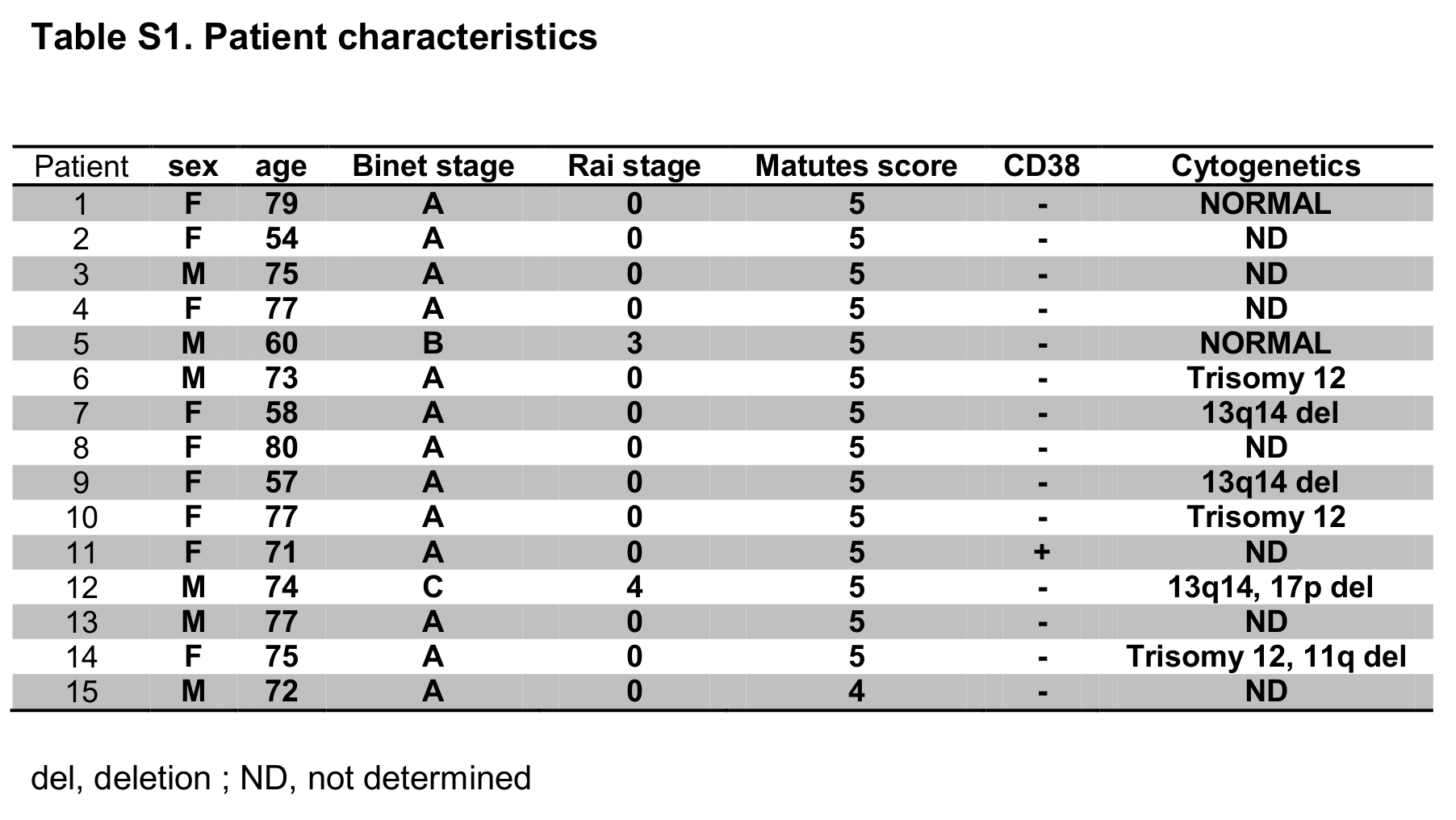

Supplement: Table S1 — Patient characteristics. (TIF) [file pone.0060370.s002.tif]

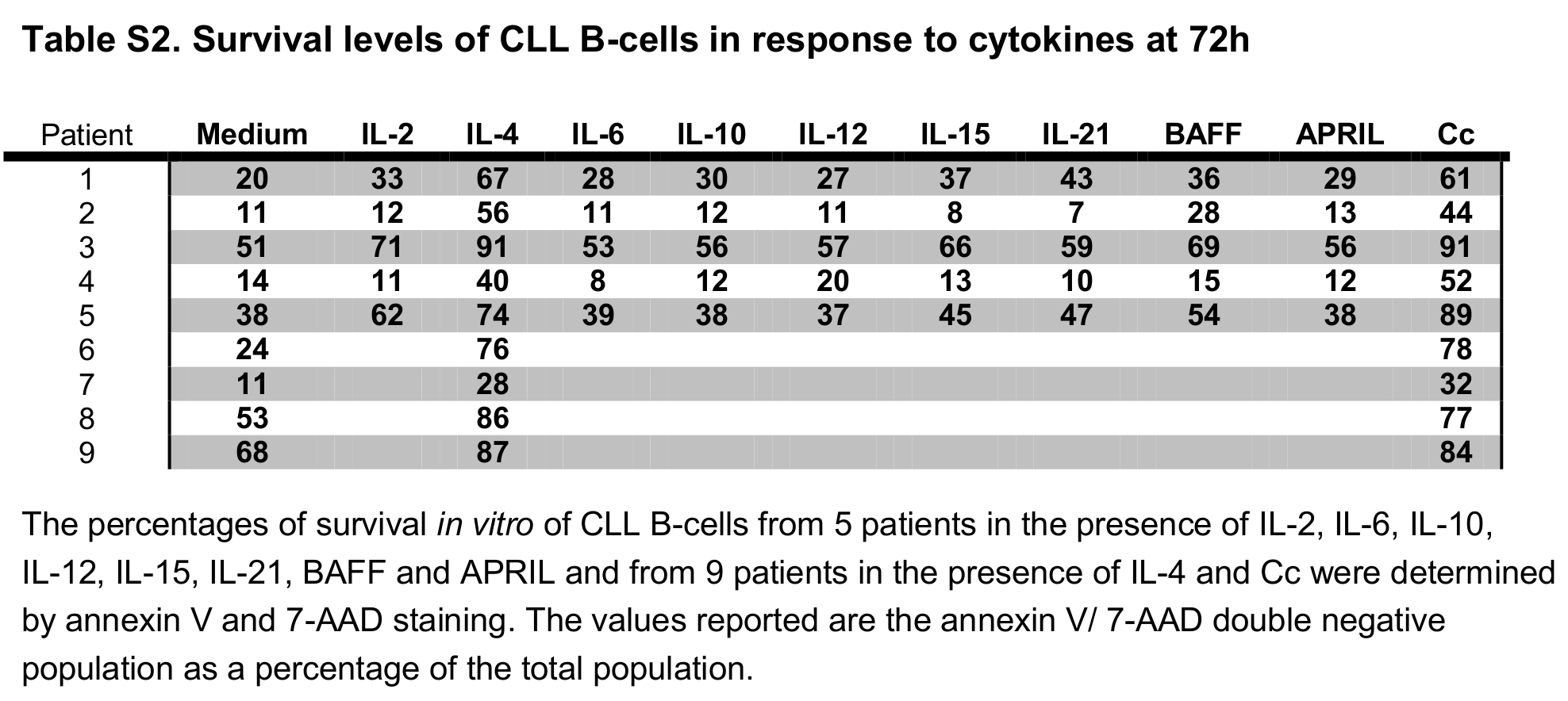

Supplement: Table S2 — Survival levels of CLL B-cells in response to cytokines at 72 h. The percentages of survival in vitro of CLL B-cells from 5 patients in the presence of IL-2, IL-6, IL-10, IL-12, IL-15, IL-21, BAFF and APRIL and from 9 patients in the presence of IL-4 and Cc were determined by annexin V and 7-AAD staining. The values reported are the annexin V/7-AAD double negative population as a percentage of the total population. (TIF) [file pone.0060370.s003.tif]
